# Pathologic responses and surgical outcomes after neoadjuvant immunochemotherapy versus neoadjuvant chemoradiotherapy in patients with locally advanced esophageal squamous cell carcinoma

**DOI:** 10.3389/fimmu.2022.1052542

**Published:** 2022-11-17

**Authors:** Lei Xu, Xiu-feng Wei, Can-jun Li, Zhao-yang Yang, Yong-kui Yu, Hao-miao Li, Hou-nai Xie, Ya-fan Yang, Wei-wei Jing, Zhen Wang, Xiao-zheng Kang, Rui-xiang Zhang, Jian-jun Qin, Li-yan Xue, Nan Bi, Xian-kai Chen, Yin Li

**Affiliations:** ^1^ Department of Thoracic Surgery, National Cancer Center/National Clinical Research Center for Cancer/Cancer Hospital, Chinese Academy of Medical Sciences and Peking Union Medical College, Beijing, China; ^2^ Department of Radiation Oncology, National Cancer Center/National Clinical Research Center for Cancer/Cancer Hospital, Chinese Academy of Medical Science and Peking Union Medical College, Beijing, China; ^3^ Department of Pathology, National Cancer Center/National Clinical Research Center for Cancer/Cancer Hospital, Chinese Academy of Medical Sciences and Peking Union Medical College, Beijing, China; ^4^ Department of Thoracic Surgery, The Affiliated Cancer Hospital of Zhengzhou University, Henan Cancer Hospital, Zhengzhou, Henan, China

**Keywords:** neoadjuvant immunotherapy, immunochemotherapy, neoadjuvant chemoradiotherapy, treatment response, postoperative complications, esophageal squamous cell carcinoma

## Abstract

**Background:**

Currently, the role of immunotherapy in neoadjuvant setting for patients with locally advanced esophageal squamous cell carcinoma (ESCC) is gradually attracting attention. Few studies compared the efficacy of neoadjuvant immunochemotherapy (NICT) and neoadjuvant chemoradiotherapy (NCRT). Our study aimed to compare treatment response and postoperative complications after NICT followed by surgery with that after conventional NCRT in patients with locally advanced ESCC.

**Methods:**

Of 468 patients with locally advanced ESCC, 154 received conventional NCRT, whereas 314 received NICT. Treatment response, postoperative complications and mortality between two groups were compared. Pathological response of primary tumor was evaluated using the Mandard tumor regression grade (TRG) scoring system. Pathological complete response (pCR) of metastatic lymph nodes (LNs) was defined as no viable tumor cell within all resected metastatic LNs. According to regression directionality, tumor regression pattern was summarized into four categories: type I, regression toward the lumen; type II, regression toward the invasive front; type III, concentric regression; and type IV, scattered regression. Inverse probability propensity score weighting was performed to minimize the influence of confounding factors.

**Results:**

After adjusting for baseline characteristics, the R0 resection rates (90.9% vs. 89.0%, P=0.302) and pCR (ypT0N0) rates (29.8% vs. 34.0%, P=0.167) were comparable between two groups. Patients receiving NCRT showed lower TRG score (P<0.001) and higher major pathological response (MPR) rate (64.7% vs. 53.6%, P=0.001) compared to those receiving NICT. However, NICT brought a higher pCR rate of metastatic LNs than conventional NCRT (53.9% vs. 37.1%, P<0.001). The rates of type I/II/III/IV regression patterns were 44.6%, 6.8%, 11.4% and 37.1% in the NICT group, 16.9%, 8.2%, 18.3% and 56.6% in the NCRT group, indicating a significant difference (P<0.001). Moreover, there were no significant differences in the incidence of total postoperative complications (35.8% vs. 39.9%, P=0.189) and 30-d mortality (0.0% vs. 1.1%, P=0.062).

**Conclusion:**

For patients with locally advanced ESCC, NICT showed a R0 resection rate and pCR (ypT0N0) rate comparable to conventional NCRT, without increased incidence of postoperative complications and mortality. Notablely, NICT followed by surgery might bring a promising treatment response of metastatic LNs.

## Introduction

Esophageal cancer (EC) is the 7th most common malignancy and the 6th leading cause of cancer mortality worldwide (1). GLOBOCAN 2020 estimated that EC represented 604,000 new cases and 544,000 deaths globally in 2020, is a major health problem (1). More than 50% of EC cases occurred in the East Asia, especially in China, and about 90% of patients have esophageal squamous cell carcinoma (ESCC) (2). ESCC is characterized by high malignancy and usually diagnosed as locally advanced disease at the first visit. In recent years, neoadjuvant therapy followed by surgery, such as neoadjuvant chemoradiotherapy (NCRT) or neoadjuvant chemotherapy (NCT), is used as a standard treatment for patients with locally advanced ESCC (3, 4). However, a recent multicenter phase III trial (NEOCRTEC5010) showed that the 5-year cumulative incidence of overall recurrence, locoregional recurrence, and distant recurrence in locally advanced ESCC patients receiving NCRT were 32.2%, 15.3%, and 24.3%, respectively, which were still unsatisfactory (3, 5). In addition, the CROSS trial reported that 35% of patients receiving NCRT experienced disease recurrence after a median follow-up of 45 months (6). Thus, it is necessary to explore a novel strategy of neoadjuvant therapy for patients with locally advanced ESCC.

Immune checkpoint inhibitors (ICIs), such as anti-programmed death 1 (PD-1) antibody, are rapidly becoming a mainstay of tumor therapy, along with surgery, chemotherapy and radiotherapy. Multiple prospective studies have demonstrated that ICIs combined with chemotherapy or chemoradiotherapy have a superior effect in the treatment of patients with advanced ESCC (7–9). Therefore, the role of ICIs in neoadjuvant setting has gained attention. Based on the result of JCOG9907 trial, NCT followed by surgery has been advocated as a choice of treatment in Asia, especially in China and Japan (10). Currently, the application and efficacy of neoadjuvant immunotherapy combined with chemotherapy is becoming the focus of clinical studies. Some single-arm studies have shown that neoadjuvant immunochemotherapy (NICT) for locally advanced ESCC produced satisfactory outcomes: a higher R0 resection rate and pathological complete response (pCR) rate and a lower toxicity profile (11–13). A multicenter, single-arm, phase II trial demonstrated that the R0 resection was achieved in 98.0% of all patients with locally advanced ESCC, and that the pCR (ypT0N0) was identified in 39.2% of all cases (12). Yang and colleagues reported that NICT induced the R0 resection rate of 100% and the pCR rate of 25% in the resected cancer specimen, and that the regimen had manageable treatment-related toxic effects during neoadjuvant therapy and did not delay surgery in patients with resectable ESCC (13). However, compared with conventional NCRT, whether NICT can be beneficial in terms of treatment response and postoperative complications and mortality is still unclear and urgently need to be investigated. Therefore, our study aimed to compare the treatment response and postoperative outcomes of NICT with that of NCRT for locally advanced ESCC. Moreover, the subgroup analysis with the inverse probability of treatment weighting (IPTW) method was performed to minimize the bias due to measured confounders.

## Patients and methods

### Patients

**Figure 1:**
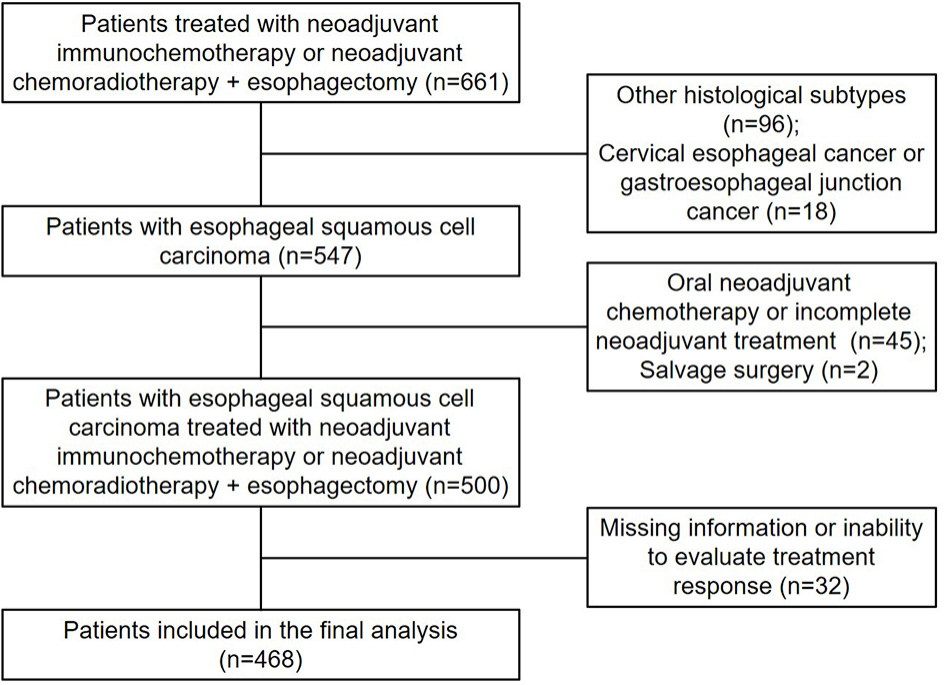
Patient selection diagram.

Our study retrospectively reviewed our prospectively collected database to identify consecutive patients who received NICT or NCRT followed by esophagectomy at Chinese National Cancer Center from June 2018 to March 2022. This retrospective study was approved by the Ethics Committee of our hospital and conducted in accordance with the Declaration of Helsinki (as revised in 2013). The informed consents were waived. Patients who meet the following inclusion criteria were included in this study: (I) Aged 18-85 years; (II) Histologically confirmed thoracic ESCC; (III) Clinical stage T1N1-3 or T2-4aN0-3 (AJCC TNM classification, 8th edition); and (IV) Karnofsky performance score (KPS): 90-100 and completed transthoracic esophagectomy followed neoadjuvant therapy. Exclusion criteria included patients receiving incomplete neoadjuvant treatment or oral chemotherapy alone or salvage surgery; patients with missing information (such as age, sex, staging and routine examinations) or inability to evaluate treatment response due to missing examination data. A total of 468 eligible patients who received NICT or NCRT followed by esophagectomy were included in this study (**Figure 1**). Of them, 314 patients were included in the NICT group, 154 patients in the NCRT group.

### Treatment protocols

Patients in the NICT group were treated with 2-4 cycles of concurrent NICT and the ICIs agents mainly consisted of camrelizumab, sintilimab, tislelizumab or pembrolizumab (200 mg Intravenous (IV) Q3W). In view of the potential impact of drug differences on the treatment response and postoperative complications, we conducted a subgroup study. The results showed that there were no statistical differences in the clinical characteristics, treatment response, postoperative complications and mortality between different immunologic drugs (**Supplement Tables 1**, **2**). All patient received at least two cycles of platinum-based two-drug combination chemotherapy, according to the latest clinical guidelines. The TP regimen comprised platinum-based drugs (cisplatin or nedaplatin: 75 mg/m^2^ IV Q3W; carboplatin: area under the curve (AUC) 5 Q3W) and paclitaxel (175 mg/m^2^ IV Q3W) or docetaxel (70 mg/m^2^ IV Q3W). The PF regimen consisted of platinum-based drugs (50 mg/m^2^ IV Q2W) and 5-fluorouracil (1000 mg/m^2^ IV Q2W). The majority of cases receiving NICT were included in some prospective trials (ChiCTR1900023880, NCCES001, PALACE-2, Keystone-002, etc.). The dosage and usage of chemotherapeutic agents and ICIs were determined based on the patient’s condition and body surface area.

Patients receiving NCRT were treated with the concurrent NCRT according to the latest clinical guidelines, and total dose of neoadjuvant radiotherapy was 32.4-50.4 Gy with 1.8 to 2.14 Gy fractions and 5 fractions per week. The dose was determined by the experienced physician for formulation of the radiotherapy plan. Gross tumor volume (GTV) was defined as any visible primary tumor identified on pre-treatment examinations (CT, EUS, MR and PET/CT, etc.), GTV-nd (metastatic regional nodes) defined as any lymph nodes diagnosed as or highly-suspected as metastatic lymph nodes. Planning gross tumor volume (PGTV) is defined as GTV and GTV-nd with 1.0cm cranial-caudal and 0.5cm lateral expansion. Clinical target volume (CTV) is defined as GTV with a 3.0-5.0 cm cranial-caudal expansion, a 0.6-0.8cm lateral expansion, and the GTV-nd with a 1.0-1.5cm expansion, including the metastatic lymph-node stations. Patients in the NCRT group received at least two cycles of platinum-based two-drug combination chemotherapy, according to the latest clinical guidelines. The TP regimen comprised platinum-based drugs (cisplatin or nedaplatin: 25 mg/m^2^ IV Q1W; carboplatin: AUC 2 Q1W) and paclitaxel (50 mg/m^2^ IV Q1W) or docetaxel (30 mg/m^2^ IV Q1W), and the PF regimen consisted of platinum-based drugs (75 mg/m^2^ IV Q3W) and 5-fluorouracil (800 mg/m^2^ IV Q3W).

Esophagectomy was usually performed within 4-8 weeks after the last cycle of neoadjuvant therapy. All patients received McKeown minimally invasive esophagectomy (MIE) combined with 2- or 3-field lymphadenectomy. The esophagus was reconstructed using the gastric tube and manual or mechanical cervical anastomosis was performed. In order to accurately assess the status of lymph nodes (LNs), complete dissection of mediastinal LNs, including bilateral laryngeal recurrent nerve LNs, was conducted for every patient. The 2-field lymphadenectomy was regularly performed and 3-field lymphadenectomy was conducted when patients with suspected positive LNs within the cervical area. All operations were performed by experienced surgeons with more than 300 cases annually, which ensured the quality of surgery.

### Pathological examination

Pathological specimens of each patient were evaluated by two experienced pathologists, mainly focusing on pathological type, resection margins, residual tumor characteristics and treatment response. R0 resection was defined as curative resection with negative resection margin (the distal, proximal or circumferential resection margin) (14). The pCR (ypT0N0) was defined as no viable tumor cells in the primary tumor area and all resected lymph nodes, major pathological response (MPR) defined as <10% residual viable tumor cells in primary tumor (14, 15). Tumor response to preoperative therapy was evaluated using the Mandard tumor regression grade (TRG) scoring system, which was assessed by the estimated proportion of residual viable tumor in relation to the original tumor area (both tumor regression changes and residual tumors), and Mandard TRG scoring system included five categories: TRG1, absence of vital residual tumor (pCR); TRG2, vital residual tumor <10% of the original tumor area; TRG3, 10%-50%; TRG4, >50%; and TRG5, absence of regressive changes (16, 17).

**Figure 2:**
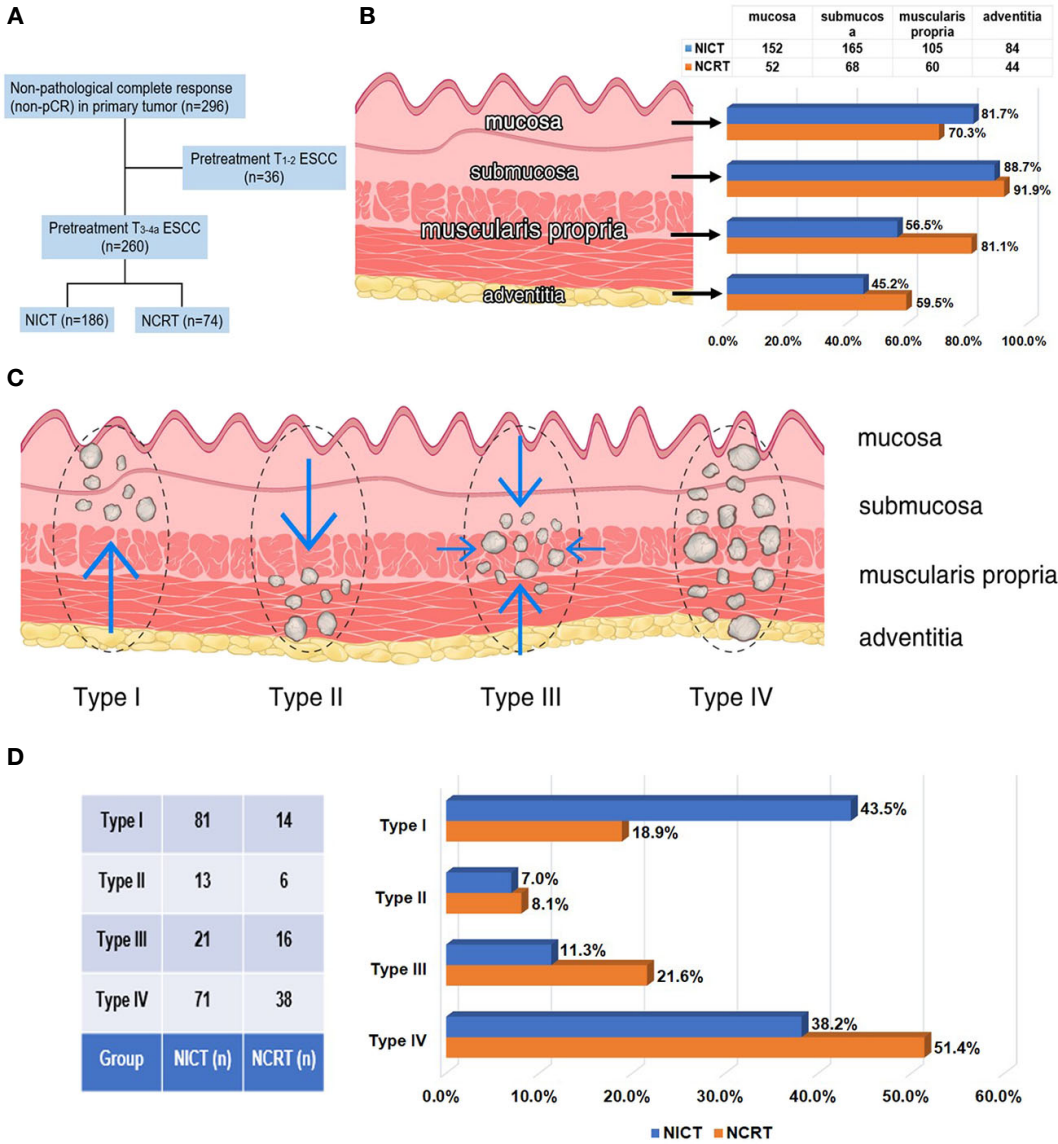
Residual tumor characteristics in 260 patients with non-pCR status of primary tumor and staged as pretreatment T3-4a after neoadjuvant therapy. **(A)** In 260 patients with non-pCR status of primary tumor and staged as pretreatment T3-4a, 186 patients received NICT and 74 patients received conventional NCRT. **(B)** Rates of cancer involvement for 4 different anatomic layers of esophageal wall in two groups. **(C)** Schematic diagram of 4 types of tumor regression patterns within the esophageal wall. **(D)** Rates of 4 different tumor regression patterns in two groups.

**Figure 3:**
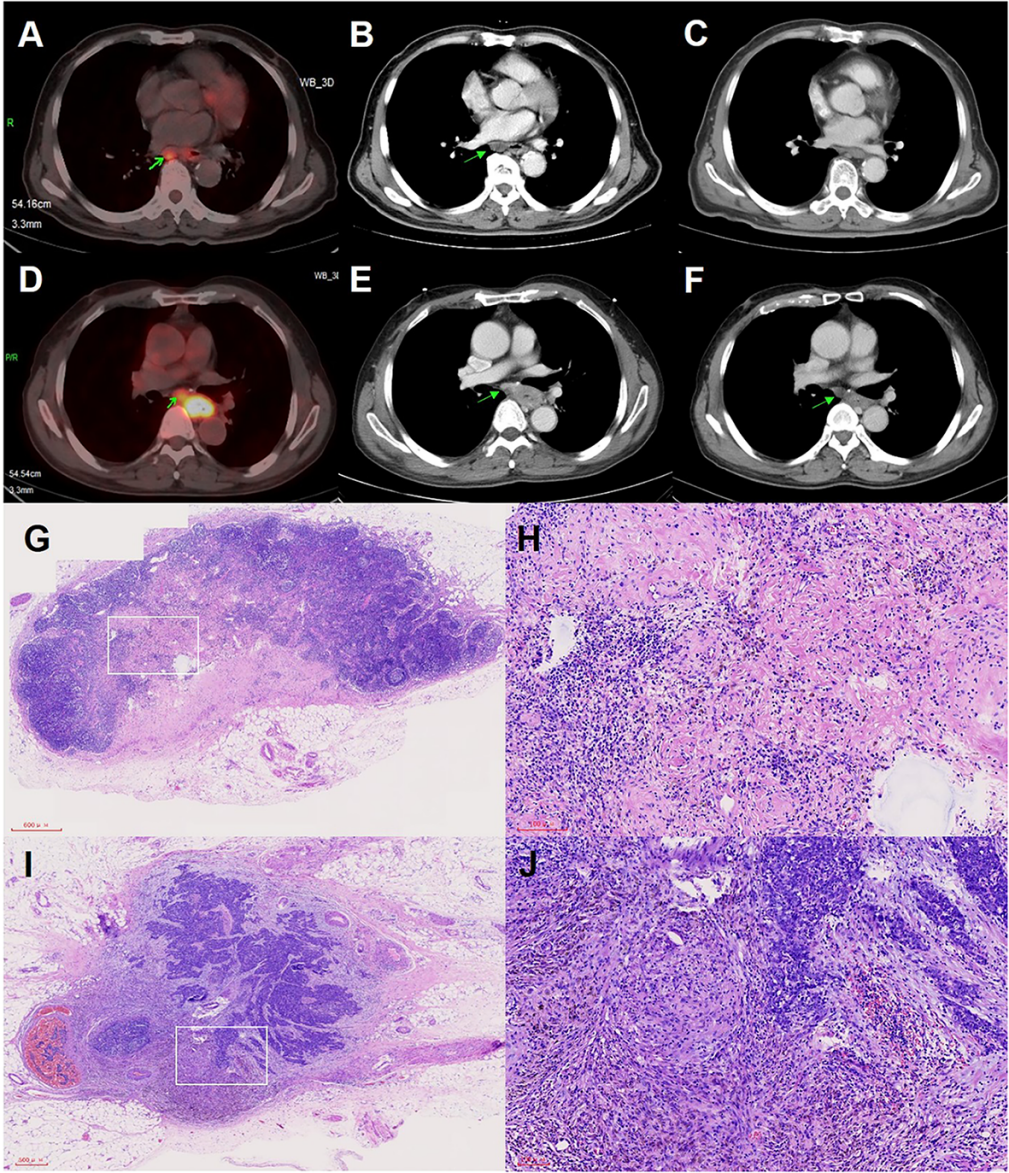
Imaging examination and pathological images of positive lymph nodes (LNs) in pCR status or non-pCR status. **(A)** PET-CT examination images of positive LNs before neoadjuvant therapy. **(B)** CT examination images of positive LNs before neoadjuvant therapy. **(C)** CT examination images of positive LNs of panel B with pCR status after neoadjuvant therapy. **(D)** PET-CT examination images of positive LNs before neoadjuvant therapy. **(E)** CT examination images of positive LNs before neoadjuvant therapy. **(F)** CT examination images of positive LNs of panel E with non-pCR status after neoadjuvant therapy. **(G)** Pathological images of positive LNs in pCR status. **(H)** Higher magnification of the white squared area of panel **(G, I)** Pathological images of positive LNs in non-pCR status. **(J)** Higher magnification of the white squared area of panel **(I)**.

In addition, we compared the directionality of tumor regression between two groups of patients. Patients with clinical stage T3-4a were included and the tumor residual pattern was summarized into four types (**Figure 2**): Type I, regression toward the lumen, residual tumors mainly in the mucosa and submucosa; Type II, regression toward the invasive front, residual tumors mainly in the muscularis propria and adventitia/surrounding stroma; Type III, concentric regression, residual tumors mainly in the submucosa and muscularis propria; and Type IV, random regression, residual tumors in all layers, as described previously (16, 18). The median number of resected LNs during lymphadenectomy was 35 (min: 10 and max: 99) and all dissected LNs underwent microscopic analysis for LN metastasis. The treatment response of metastatic LNs was evaluated based on vital residual tumor cells, necrosis, fibrosis or granulomatous changes within the nodal parenchyma. Clinically negative LNs, without evidence of regression or previous nodal involvement, were considered as ‘‘true negative LNs”. The absence of vital residual tumor cells within the nodal parenchyma, with evidence of previous tumor involvement, was defined as pCR of LNs, as shown in **Figure 3** (19).

### Postoperative complications

Morbidity and mortality within 30 days after surgery were analyzed in this study. Postoperative complications were diagnosed according to the Esophageal Complications Consensus Group (ECCG) criteria, and the severity of complications was assessed according to the Clavien-Dindo classification (20, 21). Major complication was defined as grade ≥3, in accordance with the Clavien-Dindo classification system. ICU readmission, In-hospital mortality and 30-d mortality after surgery were compared between two groups. Postoperative 30-d mortality was defined as death occurring during the first 30 days after surgery.

### Statistical analysis

The clinicopathological characteristics of two groups of patients were analyzed. Categorical variables were presented as totals and percentages, and variables between two groups were compared using the χ*^2^* test or Fisher’s exact test as appropriate. Numerical variables as mean and standard deviation (SD), the Mann-Whitney U test was used for comparative analysis. To minimize the bias due to measured confounders, the propensity score analysis with the inverse probability of treatment weighting (IPTW) method was performed (22, 23). The propensity score was calculated based on a logistic regression model, which included all covariates deemed likely to influence treatment outcomes. Statistical analyses were conducted using the R statistical software for Windows (version 3.6.0, https://cran.R-project.org). Differences were considered statistically significant at a two-sided p value <0.05.

## Results

### Patient characteristics

In our study, 154 patients received conventional NCRT, whereas 314 patients received NICT. The baseline characteristics were showed in **Table 1**. Patients in the NICT group showed lower location (P=0.022) and higher clinical N staging (P=0.032) than those in the NCRT group. However, there were no significant differences between two groups in other clinical characteristics, including age, sex, smoking index, comorbidities and clinical staging (P>0.05). After IPTW, balance in clinical characteristics between two groups was achieved, as shown in **Table 1**.

**Table 1:** Baseline characteristics of two groups of patients before and after IPTW.

Group	Level	Before IPTW			After IPTW		
		NICT (N=314)	NCRT (N=154)	P value	NICT (N=465.6)	NCRT (N=469.9)	P value
Age, year (%)	≤ 60	130 (41.4)	76 (49.4)	0.104	201.6 (43.3)	192.9 (41.1)	0.479
	> 60	184 (58.6)	78 (50.6)		264.0 (56.7)	277.0 (58.9)	
Sex (%)	male	263 (83.8)	132 (85.7)	0.584	392.6 (84.3)	391.6 (83.3)	0.699
	female	51 (16.2)	22 (14.3)		73.0 (15.7)	78.3 (16.7)	
Smoking index (%)	≥ 400	100 (31.8)	57 (37.0)	0.266	158.1 (34.0)	166.1 (35.4)	0.649
	< 400	214 (68.2)	97 (63.0)		307.5 (66.0)	303.8 (64.6)	
Comorbidities (%)	YES	144 (45.9)	74 (48.1)	0.655	215.8 (46.3)	219.0 (46.6)	0.940
	NO	170 (54.1)	80 (51.9)		249.8 (53.7)	250.9 (53.4)	
KPS (%)	90	257 (81.8)	121 (78.6)	0.398	377.6 (81.1)	383.8 (81.7)	0.818
	100	57 (18.2)	33 (21.4)		87.9 (18.9)	86.1 (18.3)	
Location (%)	upper	44 (14.0)	22 (14.3)	0.022	67.4 (14.5)	71.8 (15.3)	0.924
	middle	102 (32.5)	69 (44.8)		166.1 (35.7)	166.8 (35.5)	
	lower	168 (53.5)	63 (40.9)		232.1 (49.8)	231.3 (49.2)	
cT (%)	T1	2 (0.6)	0 (0.0)	0.113	2.0 (0.4)	0.0 (0.0)	0.591
	T2	47 (15.0)	14 (9.1)		61.4 (13.2)	68.6 (14.6)	
	T3	248 (79.0)	126 (81.8)		373.2 (80.2)	373.0 (79.4)	
	T4a	17 (5.4)	14 (9.1)		29.0 (6.2)	28.4 (6.0)	
cN (%)	N0	61 (19.4)	44 (28.6)	0.032	106.7 (22.9)	110.3 (23.5)	0.168
	N1	172 (54.8)	84 (54.5)		251.8 (54.1)	251.2 (53.5)	
	N2	76 (24.2)	26 (16.9)		102.1 (21.9)	108.4 (23.1)	
	N3	5 (1.6)	0 (0.0)		5.0 (1.1)	0.0 (0.0)	
cTNM (%)	II	98 (31.2)	54 (35.1)	0.378	151.8 (32.6)	164.1 (34.9)	0.649
	III	195 (62.1)	86 (55.8)		280.8 (60.3)	277.5 (59.1)	
	IVA	21 (6.7)	14 (9.1)		33.0 (7.1)	28.4 (6.0)	

KPS, Karnofsky performance score.

### Pathological examination

**Figure 4:**
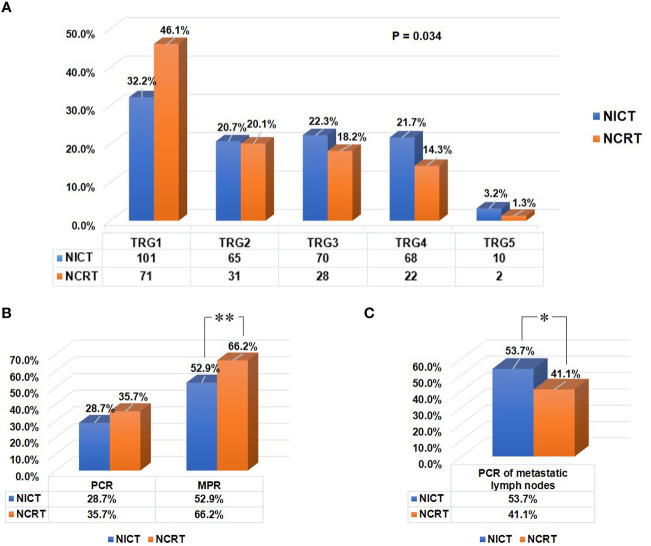
Residual tumor characteristics of all patients in both groups after neoadjuvant therapy. **(A)** The tumor regression grade (TRG) scoring in both groups. Tumor response to preoperative therapy was evaluated using the Mandard TRG scoring system. **(B)** Comparison of the pCR (ypT0N0) rates of tumor, and the MPR rates of primary tumor between two groups. **(C)** Comparison of the pCR rates of positive lymph nodes between two groups. “**“: P<0.01, “*“: P<0.05.

As shown in **Table 2**, patients receiving NICT showed comparable R0 resection rate and pCR (ypT0N0) rate to patients receiving conventional NCRT (R0 resection rate: 90.8% vs. 90.3%, P=0.860; pCR rate: 28.7% vs. 35.7%, P=0.121). Patients in the NCRT group exhibited lower pathologic T stage (P=0.001), lower TRG score (P=0.034, **Figure 4A**), and higher MPR rate (66.2% vs. 52.9%, P=0.034, **Figure 4B**), compared to those in the NICT group. However, the pCR rate of positive LNs in patients who received NICT was significantly higher than that in patients who received conventional NCRT (53.7% vs. 41.1%, P=0.040, **Figure 4C**). To analyze the regression pattern, 260 pretreatment stage T3-4a patients with residual primary tumors were included for analysis (**Figure 2A**). The number of type I - IV regression patterns in patients receiving NICT were 81 (43.5%), 13 (7.0%), 21 (11.3%), and 71 (38.2%), respectively. The number of type I - IV regression patterns in patients receiving conventional NCRT were 14 (18.9%), 6 (8.1%), 16 (21.6%), and 38 (51.4%), respectively. And there was a statistical difference in tumor regression patterns between two groups (P=0.002, **Table 2**).

**Table 2:** Pathological outcomes of two groups of patients before and after IPTW.

Group	Level	Before IPTW			After IPTW		
		NICT (N=314)	NCRT (N=154)	P value	NICT (N=465.6)	NCRT (N=469.9)	P value
R0 Resection (%)	R0	285 (90.8)	139 (90.3)	0.860	423.3 (90.9)	418.1 (89.0)	0.302
	R1	29 (9.2)	15 (9.7)		42.3 (9.1)	51.8 (11.0)	
Number of lymph nodes removed		33 ± 13	34 ± 13	0.566	33.2 ± 12.6	34.1 ± 12.6	0.494
ypT stage (%)	T0	101 (32.2)	71 (46.1)	0.001	148.2 (31.8)	217.7 (46.3)	< 0.001
	T1	83 (26.4)	18 (11.7)		126.3 (17.2)	52.8 (11.3)	
	T2	47 (15.0)	23 (14.9)		69.6 (15.0)	63.9 (13.6)	
	T3	81 (25.8)	41 (26.6)		118.9 (25.5)	132.9 (28.3)	
	T4a	2 (0.6)	1 (0.6)		2.5 (0.5)	2.6 (0.6)	
ypN stage (%)	N0	196 (62.4)	101 (65.6)	0.521	298.4 (64.1)	280.2 (59.6)	0.122
	N1	67 (21.3)	35 (22.7)		97.1 (20.9)	126.4 (26.9)	
	N2	37 (11.8)	15 (9.7)		50.2 (10.8)	50.7 (10.8)	
	N3	14 (4.5)	3 (1.9)		19.9 (4.3)	12.6 (2.7)	
ypTNM stage (%)	I	169 (53.8)	81 (52.6)	0.491	257.9 (55.4)	222.6 (47.4)	0.059
	II	26 (8.3)	18 (11.7)		39.2 (8.4)	49.9 (10.6)	
	III	104 (33.1)	51 (33.1)		147.3 (31.6)	179.7 (38.3)	
	IVA	15 (4.8)	4 (2.6)		21.1 (4.5)	17.7 (3.8)	
PCR (%)	YES	90 (28.7)	55 (35.7)	0.121	138.6 (29.8)	159.7 (34.0)	0.167
	NO	224 (71.3)	99 (64.3)		327.0 (70.2)	310.2 (66.0)	
MPR (%)	YES	166 (52.9)	102 (66.2)	0.006	249.7 (53.6)	304.1 (64.7)	0.001
	NO	148 (47.1)	52 (33.8)		215.9 (46.4)	165.8 (35.3)	
TRG score (%)	TRG1	101 (32.2)	71 (46.1)	0.034	148.2 (31.8)	217.7 (46.3)	< 0.001
	TRG2	65 (20.7)	31 (20.1)		101.5 (21.8)	86.3 (18.4)	
	TRG3	70 (22.3)	28 (18.2)		105.4 (22.6)	76.9 (16.4)	
	TRG4	68 (21.7)	22 (14.3)		95.8 (20.6)	78.6 (16.7)	
	TRG5	10 (3.2)	2 (1.3)		14.7 (3.2)	10.4 (2.2)	
Residual tumor pattern (%)	I	81 (43.5)	14 (18.9)	0.002	124.8 (44.6)	36.5 (16.9)	< 0.001
	II	13 (7.0)	6 (8.1)		19.2 (6.8)	18.1 (8.2)	
	III	21 (11.3)	16 (21.6)		32.0 (11.4)	39.6 (18.3)	
	IV	71 (38.2)	38 (51.4)		104.1 (37.1)	123.6 (56.6)	
PCR of LNM (%)	YES	137 (53.7)	37 (41.1)	0.040	195.0 (53.9)	111.5 (37.1)	< 0.001
	NO	118 (46.3)	53 (58.9)		167.2 (46.1)	189.7 (62.9)	

PCR, Pathological complete response; MPR, Major pathological response; TRG, Tumor regression grade; LNM, Lymph node metastasis.

After balancing the baseline characteristics, similar results were observed. There was no statistical difference in R0 resection rate between two groups (90.9% vs. 89.0%, P=0.302), and the pCR (ypT0N0) rates were comparable (29.8% vs. 34.0%, P=0.167). Patients in the NCRT group exhibited lower pathologic T stage (P<0.001), lower TRG score (P<0.001), and higher MPR rate (64.7% vs. 53.6%, P=0.001), compared to those in the NICT group. However, NICT brought a higher pCR rate of positive LNs than conventional NCRT (53.9% vs. 37.1%, P<0.001). The rates of type I/II/III/IV regression patterns were 44.6%, 6.8%, 11.4% and 37.1% in the NICT group, 16.9%, 8.2%, 18.3% and 56.6% in the NCRT group, indicating a significant difference (P<0.001, **Table 2**). However, there were no significant differences in other pathological variables (P>0.05).

### Postoperative complications and mortality

Between two groups, there were no significant differences in the incidence of total postoperative complications (35.4% vs. 39.0%, P=0.446, **Table 3**), major postoperative complications (9.9% vs. 14.3%, P=0.157) and pulmonary complications (21.0% vs. 22.1%, P=0.793). Two groups of patients also showed similar Clavien-Dindo grades (P=0.363), as shown in **Table 3**. Moreover, there were no significant differences in ICU readmission (3.2% vs. 6.5%, P=0.096) and 30-d mortality (0.0% vs. 1.3%, P=0.108). In NCRT group, one patient died of postoperative respiratory failure within 30 days after surgery, and the other died of postoperative tracheoesophageal leakage. After IPTW, similar results were observed. There were no significant differences in the incidence of total postoperative complications (35.8% vs. 39.9%, P=0.189, **Table 3**), major postoperative complications (10.6% vs. 14.2%, P=0.085) and pulmonary complications (21.7% vs. 24.3%, P=0.348). And two groups of patients also showed similar Clavien-Dindo grades (P=0.079). Additionally, there were no significant differences in ICU readmission (3.4% vs. 5.7%, P=0.091) and 30-d mortality (0.0% vs. 1.1%, P=0.062).

**Table 3:** Postoperative complications and mortality of two groups of patients before and after IPTW.

Group	Before IPTW			After IPTW		
	NICT (N=314)	NCRT (N=154)	P value	NICT (N=465.6)	NCRT (N=469.9)	P value
Total postoperative complications (%)	111 (35.4)	60 (39.0)	0.446	166.6 (35.8)	187.6 (39.9)	0.189
Major postoperative complications (%)	31 (9.9)	22 (14.3)	0.157	49.3 (10.6)	66.6 (14.2)	0.085
Anastomotic leak (%)	9 (2.9)	7 (4.5)	0.348	13.6 (2.9)	20.2 (4.3)	0.306
RLN injury (%)	8 (2.5)	6 (3.9)	0.421	10.5 (2.3)	14.2 (3.0)	0.558
Pulmonary complications (%)	66 (21.0)	34 (22.1)	0.793	101.0 (21.7)	114.1 (24.3)	0.348
Pneumonia (%)	34 (10.8)	18 (11.7)	0.781	34 (10.8)	18 (11.7)	0.400
Pneumothorax (%)	7 (2.2)	5 (3.2)	0.513	9.7 (2.1)	15.7 (3.3)	0.241
Atelectasis (%)	2 (0.6)	2 (1.3)	0.601	3.3 (0.7)	13.8 (2.3)	0.056
Pleural effusions (%)	27 (8.6)	14 (9.1)	0.860	42.5 (9.1)	45.1 (9.6)	0.856
Respiratory failure (%)	3 (1.0)	5 (3.2)	0.122	4.6 (1.0)	14.2 (3.0)	0.061
Cardiac complications (%)	17 (5.4)	13 (8.4)	0.209	25.1 (5.4)	36.5 (7.8)	0.123
Other complications (%)	20 (6.4)	16 (10.4)	0.125	29.8 (6.4)	43.1 (9.2)	0.122
Clavien-Dindo grade (%)			0.363			0.079
I	41 (36.9)	20 (33.3)		59.1 (35.5)	60.7 (32.6)	
II	39 (35.1)	18 (30.0)		58.2 (34.7)	60.3 (32.1)	
III	25 (22.5)	14 (23.3)		39.5 (24.0)	38.0 (20.3)	
IV	6 (5.4)	7 (11.7)		9.8 (6.0)	26.2 (13.9)	
V	0 (0.0)	1 (1.7)		0.0 (0.0)	2.3 (1.1)	
ICU readmission (%)	10 (3.2)	10 (6.5)	0.096	15.9 (3.4)	27.1 (5.7)	0.091
In-hospital mortality (%)	0 (0.0)	0 (0.0)	–	0 (0.0)	0 (0.0)	–
30-d mortality (%)	0 (0.0)	2 (1.3)	0.108	0.0 (0.0)	5.2 (1.1)	0.062

RLN, Recurrent laryngeal nerve; ICU: intensive care unit.

## Discussion

In our study, NICT showed a R0 resection rate and pCR (ypT0N0) rate comparable to NCRT, without increasing incidence of postoperative complications and mortality. Patients in the NCRT group exhibited higher MPR rate and lower TRG score, compared to those in the NICT group. However, patients who received NICT had significantly higher pCR rate of positive LNs than those who received conventional NCRT. Furthermore, it is worth noting that two groups of patients showed different regression patterns of primary tumor. To minimize the influence of potential confounders, the IPTW analysis was performed. After balancing the baseline characteristics, similar results were observed. Therefore, as a novel treatment option, NICT followed by surgery showed a good performance in term of some treatment response.

A meta-analysis, including multiple clinical studies, reported an MPR rate of 53.5% (95%CI: 47.9%–59%) in patients who underwent NICT (15). Our study observed an MPR rate (52.9%) in the NICT group, similar to the meta-analysis. However, patients in the NCRT group showed higher MPR rate and lower TRG score compared to those in the NICT group. This may be due to the better performance of radiotherapy in the control of the primary tumor in the NCRT group. Furthermore, the pCR of positive LNs after neoadjuvant therapy is considered as an important predictor for better prognosis (19, 24, 25). The pCR rate of positive LNs was reported to be 30.0% in patients receiving NCT and 29.2% in those receiving NCRT (18, 19). Our study found that the pCR rate of positive LNs in patients receiving NICT was significantly higher than that in patients receiving conventional NCRT (53.7% vs. 41.1%). Additionally, our study showed discordance in pCR between the primary tumor and positive LNs, consistent with the results reported previously (19). Pathological LN response was considered to be a better predictor of long-term survival than primary tumor response, and patients with pathological LN complete response were demonstrated to have similar overall survival and cumulative incidence of recurrences as those with pretreatment negative LNs (19, 25). Overall, the pCR of positive LNs was one of the most important indicators in patients receiving neoadjuvant therapy, and NICT showed a better performance in this indicator compared with conventional NCRT.

Several single-arm clinical studies reported R0 resection rates of 80.5%-98.0% in ESCC patients receiving NICT (12, 13, 26). In our study, patients in the NICT group had a similar R0 resection rate (90.8%), and this group of patients showed an R0 resection rate comparable to patients receiving conventional NCRT. Moreover, we obtained that the pCR (ypT0N0) rates were similar between two groups (28.7% vs. 35.7%, P=0.121). A multicenter randomized clinical trial demonstrated that neoadjuvant chemotherapy (NCT) generated lower R0 resection rate (74% vs. 87%, P=0.04) and pCR rate (9% vs. 28%, P=0.002) compared to NCRT (27). However, NICT was proven to bring higher pCR rate (41.7% vs. 10.7%) than NCT (28). It could be seen that the addition of immunotherapy to chemotherapy might improve the treatment response of tumors and surgical results. Based on our preliminary experience, NCRT has a better control rate of primary tumor (higher MPR rate and lower TRG score in primary tumor), while NICT performs better in treatment response of metastatic lymph node. so that patients could obtain a R0 resection rate and pCR (ypT0N0) rate that are comparable to those of patients receiving NCRT.

It was worth noting that diverse modes of residual tumor distribution were observed between two groups. Type IV was considered significantly more frequent than other types after receiving NCRT (16), and consistent results were observed in our study. However, the type I regression pattern was significantly more common after receiving NICT in our study. Tumor regression patterns may be determined by treatment strategies and tumor-specific natures, including intratumoral heterogeneity and tumor microenvironment. The heterogeneity could be caused by mutations, copy number variations and single nucleotide polymorphisms, and tumor microenvironment involved the interaction between tumor cells and the extracellular matrix, immune cells, and fibroblasts (29, 30). Their critical roles had been demonstrated in determining the sensitivity of tumor cells to immunotherapy or chemoradiotherapy (30, 31). The differential response of tumor cells with tumor specific natures to various treatment strategies may lead to the difference in tumor residual patterns between two groups. Currently, little is known about the features of residual tumor distribution after neoadjuvant therapy. Therefore, the impact of tumor residual patterns on metastasis and prognosis is still unknown, and further studies are needed.

The postoperative mortality and morbidity were comparable between the NICT group and the NCRT group, consistent with previous studies (5). Hong et al. (5) compared major postoperative complications and 30-d mortality between patients receiving NICT and NCRT, and found that patients receiving NICT had similar incidences of major complications and 30-d mortality, compared to those receiving NCRT. In addition, previous studies confirmed that the addition of immunotherapy to NCT did not significantly increase the risk of developing postoperative pulmonary complications and mortality compared with NCT, and that immunotherapy combined with NCRT was not associated with significantly increased incidence of major postoperative complications (including pulmonary complications, anastomotic leakage and other complications) compared to NCRT (28, 32– 34). These results indicated that the addition of immunotherapy did not increase the risk of surgery and the incidence of related postoperative complications.

This study had several limitations. Firstly, this study had a retrospective design, which is inherently biased. To minimize the influence of potential confounding factors and eliminate selection bias, we performed the IPTW-adjusted analysis, which has some potential advantages over more common matching techniques (propensity score matching, PSM), such as retaining all the samples. Secondly, the regimens and doses of ICIs and chemotherapy drugs were not exactly the same, which might potentially affect our results. In addition, there were patients who treated with radiation dose lower than 40Gy because of suspected complications (such as esophageal perforation) caused by radiotherapy in the NCRT group, which might have some influence on treatment outcomes (pCR rate or R0 resection rate). However, the proportion of these patients was very small, and promising treatment results had been observed in a certain proportion of these population. Finally, there were deficiencies in the clinical diagnoses of positive LNs. While PET/CT, EUS, CT and/or MRI were included in the routine examinations, EUS-fine needle aspiration was not used to obtain tissue proof for each suspicious lymph node. In our study, surgical specimens of every lymph node were evaluated to find evidence of regression or previous nodal involvement, which could be used to reconfirm the clinical diagnosis of positive LNs.

## Conclusions

In conclusion, for patients with locally advanced thoracic ESCC, NICT followed by surgery showed a R0 resection rate and pCR (ypT0N0) rate comparable to conventional NCRT followed by surgery, without increasing incidence of postoperative complications and mortality. However, NICT had significant pathological advantages compared to conventional NCRT, especially a higher pCR rate of positive LNs. Moreover, patients receiving NICT also showed different regression patterns of primary tumors. Therefore, as a novel therapeutic option, NICT followed by surgery may result in a promising R0 resection rate and pCR (ypT0N0) rate, shows a better performance in therapeutic response of metastatic LNs for patients with locally advanced ESCC.

## Data availability statement

The raw data supporting the conclusions of this article will be made available by the authors, without undue reservation.

## Ethics statement

The studies involving human participants were reviewed and approved by the Ethics Committee of Chinese National Cancer Center. The ethics committee waived the requirement of written informed consent for participation.

## Author contributions

Conception and design: All authors.Provision of study materials or patients: LX, X-FW, C-JL, Z-YY, H-NX, Y-FY, ZW, X-ZK, R-XZ, L-YX, X-KC, and YL. Collection and assembly of data: LX, X-FW, C-JL, Z-YY, H-NX, Y-KY, Y-FY, ZW, X-ZK, L-YX, X-KC, and YL. Data analysis and interpretation: All authors. Manuscript writing: All authors. Final approval of manuscript: All authors. Accountable for all aspects of the work: All authors. All authors contributed to the article and approved the submitted version.

## Funding

This study was funded by the Special Program for Basic Resource Survey of the Ministry of Science and Technology (2019FY101101).

## Acknowledgments

We are very grateful to Jie-yu Zhang for her assistance with the schematic diagram, and to Ling-hong Kong for support in pathological evaluation.

## Conflict of interest

The authors declare that the research was conducted in the absence of any commercial or financial relationships that could be construed as a potential conflict of interest.

## Publisher’s note

All claims expressed in this article are solely those of the authors and do not necessarily represent those of their affiliated organizations, or those of the publisher, the editors and the reviewers. Any product that may be evaluated in this article, or claim that may be made by its manufacturer, is not guaranteed or endorsed by the publisher.

## References

[1] Sung H Ferlay J Siegel RL Laversanne M Soerjomataram I Jemal A. Global cancer statistics 2020: GLOBOCAN estimates of incidence and mortality worldwide for 36 cancers in 185 countries. CA Cancer J Clin (2021) 71(3):209–49. doi: 10.3322/caac.21660 33538338

[2] Chen W Zheng R Baade PD Zhang S Zeng H Bray F. Cancer statistics in China, 2015. CA Cancer J Clin (2016) 66(2):115–32. doi: 10.3322/caac.21338 26808342

[3] Yang H Liu H Chen Y Zhu C Fang W Yu Z. Neoadjuvant chemoradiotherapy followed by surgery versus surgery alone for locally advanced squamous cell carcinoma of the esophagus (NEOCRTEC5010): A phase III multicenter, randomized, open-label clinical trial. J Clin Oncol (2018) 36(27):2796–803. doi: 10.1200/JCO.2018.79.1483 PMC6145832 30089078

[4] Wong IYH Lam KO Zhang RQ Chan WWL Wong CLY Chan FSY. Neoadjuvant chemoradiotherapy using cisplatin and 5-fluorouracil (PF) versus carboplatin and paclitaxel (CROSS regimen) for esophageal squamous cell carcinoma (ESCC): A propensity score-matched study. Ann Surg (2020) 272(5):779–85. doi: 10.1097/SLA.0000000000004329 32833766

[5] Hong ZN Gao L Weng K Huang Z Han W Kang M. Safety and feasibility of esophagectomy following combined immunotherapy and chemotherapy for locally advanced esophageal squamous cell carcinoma: A propensity score matching analysis. Front Immunol (2022) 13:836338. doi: 10.3389/fimmu.2022.836338 35300335 PMC8921090

[6] Oppedijk V van der Gaast A van Lanschot JJ van Hagen P van Os R van Rij CM. Patterns of recurrence after surgery alone versus preoperative chemoradiotherapy and surgery in the CROSS trials. J Clin Oncol (2014) 32(5):385–91. doi: 10.1200/JCO.2013.51.2186 24419108

[7] Zhang B Qi L Wang X Xu J Liu Y Mu L. Phase II clinical trial using camrelizumab combined with apatinib and chemotherapy as the first-line treatment of advanced esophageal squamous cell carcinoma. Cancer Commun (Lond) (2020) 40(12):711–20. doi: 10.1002/cac2.12119 PMC7743020 33314747

[8] Doki Y Ajani JA Kato K Xu J Wyrwicz L Motoyama S. Nivolumab combination therapy in advanced esophageal squamous-cell carcinoma. N Engl J Med (2022) 386(5):449–62. doi: 10.1056/NEJMoa2111380 35108470

[9] Luo H Lu J Bai Y Mao T Wang J Fan Q. Effect of camrelizumab vs placebo added to chemotherapy on survival and progression-free survival in patients with advanced or metastatic esophageal squamous cell carcinoma: The ESCORT-1st randomized clinical trial. JAMA (2021) 326(10):916–25. doi: 10.1001/jama.2021.12836 PMC8441593 34519801

[10] Ando N Kato H Igaki H Shinoda M Ozawa S Shimizu H. A randomized trial comparing postoperative adjuvant chemotherapy with cisplatin and 5-fluorouracil versus preoperative chemotherapy for localized advanced squamous cell carcinoma of the thoracic esophagus (JCOG9907). Ann Surg Oncol (2012) 19(1):68–74. doi: 10.1245/s10434-011-2049-9 21879261

[11] Yan X Duan H Ni Y Zhou Y Wang X Qi H. Tislelizumab combined with chemotherapy as neoadjuvant therapy for surgically resectable esophageal cancer: A prospective, single-arm, phase II study (TD-NICE). Int J Surg (2022) 103:106680. doi: 10.1016/j.ijsu.2022.106680 35595021

[12] Liu J Yang Y Liu Z Fu X Cai X Li H. Multicenter, single-arm, phase II trial of camrelizumab and chemotherapy as neoadjuvant treatment for locally advanced esophageal squamous cell carcinoma. J Immunother Cancer. (2022) 10(3):e004291. doi: 10.1136/jitc-2021-004291 35338088 PMC8961177

[13] He W Leng X Mao T Luo X Zhou L Yan J. Toripalimab plus paclitaxel and carboplatin as neoadjuvant therapy in locally advanced resectable esophageal squamous cell carcinoma. Oncologist (2022) 27(1):e18–28. doi: 10.1093/oncolo/oyab011 PMC8842349 35305102

[14] Duan H Shao C Pan M Liu H Dong X Zhang Y. Neoadjuvant pembrolizumab and chemotherapy in resectable esophageal cancer: An open-label, single-arm study (PEN-ICE). Front Immunol (2022) 13:849984. doi: 10.3389/fimmu.2022.849984 35720388 PMC9202755

[15] Wang Z Shao C Wang Y Duan H Pan M Zhao J. Efficacy and safety of neoadjuvant immunotherapy in surgically resectable esophageal cancer: A systematic review and meta-analysis. Int J Surg (2022) 104:106767. doi: 10.1016/j.ijsu.2022.106767 35840049

[16] Tang H Jiang D Zhang S Zeng Z Tan L Hou Y. Residual tumor characteristics of esophageal squamous cell carcinoma after neoadjuvant chemoradiotherapy. J Thorac Cardiovasc Surg (2020) 162(6):S0022–5223(20)32634-9. doi: 10.1016/j.jtcvs.2020.09.042 33268125

[17] Mandard AM Dalibard F Mandard JC Marnay J Henry-Amar M Petiot JF. Pathologic assessment of tumor regression after preoperative chemoradiotherapy of esophageal carcinoma. Clinicopathologic correlations. Cancer. (1994) 73(11):2680–6. doi: 10.1002/1097-0142(19940601)73:11<2680::aid-cncr2820731105>3.0.co;2-c 8194005

[18] Shapiro J ten Kate FJ van Hagen P Ruurda JP. Residual esophageal cancer after neoadjuvant chemoradiotherapy frequently involves the mucosa and submucosa. Ann Surg (2013) 258(5):678–88. doi: 10.1097/SLA.0b013e3182a6191d 24096766

[19] Hagi T Makino T Yamasaki M Yamashita K Tanaka K Saito T. Pathological regression of lymph nodes better predicts long-term survival in esophageal cancer patients undergoing neoadjuvant chemotherapy followed by surgery. Ann Surg (2022) 275(6):1121–9. doi: 10.1097/SLA.0000000000004238 PMC10060043 32910622

[20] Low DE Alderson D Cecconello I Chang AC Darling GE DʼJourno XB. International consensus on standardization of data collection for complications associated with esophagectomy: Esophagectomy complications consensus group (ECCG). Ann Surg (2015) 262(2):286–94. doi: 10.1097/SLA.0000000000001098 25607756

[21] Dindo D Demartines N Clavien PA. Classification of surgical complications: a new proposal with evaluation in a cohort of 6336 patients and results of a survey. Ann Surg (2004) 240(2):205–13. doi: 10.1097/01.sla.0000133083.54934.ae PMC1360123 15273542

[22] Austin PC Stuart EA. Moving towards best practice when using inverse probability of treatment weighting (IPTW) using the propensity score to estimate causal treatment effects in observational studies. Stat Med (2015) 34(28):3661–79. doi: 10.1002/sim.6607 PMC4626409 26238958

[23] Reifeis SA Hudgens MG. On variance of the treatment effect in the treated when estimated by inverse probability weighting. Am J Epidemiol (2022) 191(6):1092–7. doi: 10.1093/aje/kwac014 PMC9271225 35106534

[24] Guo X Wang Z Yang H Mao T Chen Y Zhu C. Impact of lymph node dissection on survival after neoadjuvant chemoradiotherapy for locally advanced esophageal squamous cell carcinoma: From the results of NEOCRTEC5010, a randomized multicenter study. Ann Surg (2021). doi: 10.1097/SLA.0000000000004798 33605586

[25] Hsu PK Yeh YC Chien LI Huang CS Hsu HS. Clinicopathological significance of pathologic complete lymph node regression after neoadjuvant chemoradiotherapy in esophageal squamous cell carcinoma. Ann Surg Oncol (2021) 28(4):2048–58. doi: 10.1245/s10434-020-09363-z 33216266

[26] Wu Z Zheng Q Chen H Xiang J Hu H Li H. Efficacy and safety of neoadjuvant chemotherapy and immunotherapy in locally resectable advanced esophageal squamous cell carcinoma. J Thorac Dis (2021) 13(6):3518–28. doi: 10.21037/jtd-21-340 PMC8264718 34277047

[27] Klevebro F Alexandersson von Döbeln G Wang N Johnsen G Jacobsen AB Friesland S. A randomized clinical trial of neoadjuvant chemotherapy versus neoadjuvant chemoradiotherapy for cancer of the oesophagus or gastro-oesophageal junction. Ann Oncol (2016) 27(4):660–7. doi: 10.1093/annonc/mdw010 26782957

[28] Qiao Y Zhao C Li X Zhao J Huang Q Ding Z. Efficacy and safety of camrelizumab in combination with neoadjuvant chemotherapy for ESCC and its impact on esophagectomy. Front Immunol (2022) 13:953229. doi: 10.3389/fimmu.2022.953229 35911723 PMC9329664

[29] Gao YB Chen ZL Li JG Hu XD Shi XJ Sun ZM. Genetic landscape of esophageal squamous cell carcinoma. Nat Genet (2014) 46(10):1097–102. doi: 10.1038/ng.3076 25151357

[30] Hanoteau A Newton JM Krupar R Huang C Liu HC Gaspero A. Tumor microenvironment modulation enhances immunologic benefit of chemoradiotherapy. J Immunother Cancer. (2019) 7(1):10. doi: 10.1186/s40425-018-0485-9 30646957 PMC6332704

[31] Pitt JM Marabelle A Eggermont A Soria JC Kroemer G Zitvogel L. Targeting the tumor microenvironment: removing obstruction to anticancer immune responses and immunotherapy. Ann Oncol (2016) 27(8):1482–92. doi: 10.1093/annonc/mdw168 27069014

[32] Park SY Hong MH Kim HR Lee CG Cho JH Cho BC. The feasibility and safety of radical esophagectomy in patients receiving neoadjuvant chemoradiotherapy with pembrolizumab for esophageal squamous cell carcinoma. J Thorac Dis (2020) 12(11):6426–34. doi: 10.21037/jtd-20-1088 PMC7711420 33282345

[33] Sihag S Ku GY Tan KS Nussenzweig S Wu A Janjigian YY. Safety and feasibility of esophagectomy following combined immunotherapy and chemoradiotherapy for esophageal cancer. J Thorac Cardiovasc Surg 161(3):836-43.e1. doi: 10.1016/j.jtcvs.2020.11.106 PMC7889638 33485662

[34] Shen D Chen Q Wu J Li J Tao K Jiang Y. The safety and efficacy of neoadjuvant PD-1 inhibitor with chemotherapy for locally advanced esophageal squamous cell carcinoma. J Gastrointest Oncol (2021) 12(1):1–10. doi: 10.21037/jgo-20-599 33708420 PMC7944149

