# Supplementary material for: Pathologic responses and surgical outcomes after neoadjuvant immunochemotherapy versus neoadjuvant chemoradiotherapy in patients with locally advanced esophageal squamous cell carcinoma
Source: Front Immunol. 2022 Nov 17;13:1052542. doi: 10.3389/fimmu.2022.1052542 (PMC9713810; doi:10.3389/fimmu.2022.1052542)
Supplement: Supplementary file 1 [file Table_1.docx]

**Supplement Table 1. Baseline characteristics of patients receiving each ICIs.**

| Group | Level | Camrelizumab (N=178) | Pembrolizumab (N=69) | Sintilimab (N=39) | Tislelizumab (N=28) | P value |
| --- | --- | --- | --- | --- | --- | --- |
| Age, year (%) | ≤ 60 | 71 (39.9) | 31 (44.9) | 13 (33.3) | 15 (53.6) | 0.351 |
|  | > 60 | 107 (60.1) | 38 (55.1) | 26 (66.7) | 13 (46.4) |  |
| Sex (%) | male | 150 (84.3) | 56 (81.2) | 32 (82.1) | 25 (89.3) | 0.780 |
|  | female | 28 (15.7) | 13 (18.8) | 7 (17.9) | 3 (10.7) |  |
| Smoking index (%) | ≥ 400 | 56 (31.5) | 25 (36.2) | 13 (33.3) | 6 (21.4) | 0.559 |
|  | < 400 | 122 (68.5) | 44 (63.8) | 26 (66.7) | 22 (78.6) |  |
| Comorbidities (%) | YES | 82 (46.1) | 33 (47.8) | 14 (35.9) | 15 (53.6) | 0.505 |
|  | NO | 96 (53.9) | 36 (52.2) | 25 (64.1) | 13 (46.4) |  |
| KPS (%) | 90 | 146 (82.0) | 54 (78.3) | 32 (82.1) | 25 (89.3) | 0.649 |
|  | 100 | 32 (18.0) | 15 (21.7) | 7 (17.9) | 3 (10.7) |  |
| Location (%) | upper | 24 (13.5) | 10 (14.5) | 5 (12.8) | 5 (17.9) | 0.910 |
|  | middle | 59 (33.1) | 25 (36.2) | 10 (25.6) | 8 (28.6) |  |
|  | lower | 95 (53.4) | 34 (49.3) | 24 (61.5) | 15 (53.6) |  |
| cT (%) | T1 | 0 (0.0) | 1 (1.4) | 0 (0.0) | 1 (3.6) | 0.216 |
|  | T2 | 20 (11.2) | 15 (21.7) | 7 (17.9) | 5 (17.9) |  |
|  | T3 | 148 (83.1) | 49 (71.0) | 31 (79.5) | 20 (71.4) |  |
|  | T4a | 10 (5.6) | 4 (5.8) | 1 (2.6) | 2 (7.1) |  |
| cN (%) | N0 | 29 (16.3) | 14 (20.3) | 11 (28.2) | 7 (25.0) | 0.531 |
|  | N1 | 101 (56.7) | 36 (52.2) | 21 (53.8) | 14 (50.0) |  |
|  | N2 | 43 (24.2) | 19 (27.5) | 7 (17.9) | 7 (25.0) |  |
|  | N3 | 5 (2.8) | 0 (0.0) | 0 (0.0) | 0 (0.0) |  |
| cTNM (%) | II | 46 (25.8) | 23 (33.3) | 18 (46.2) | 11 (39.3) | 0.227 |
|  | III | 118 (66.3) | 42 (60.9) | 20 (51.3) | 15 (53.6) |  |
|  | IVA | 14 (7.9) | 4 (5.8) | 1 (2.6) | 2 (7.1) |  |

KPS, Karnofsky performance score.
