# Supplementary material for: Pathologic responses and surgical outcomes after neoadjuvant immunochemotherapy versus neoadjuvant chemoradiotherapy in patients with locally advanced esophageal squamous cell carcinoma
Source: Front Immunol. 2022 Nov 17;13:1052542. doi: 10.3389/fimmu.2022.1052542 (PMC9713810; doi:10.3389/fimmu.2022.1052542)
Supplement: Supplementary file 2 [file Table_2.docx]

**Supplement Table 2. Surgical and pathological outcomes of patients receiving each ICIs.**

| Group | Level | Camrelizumab (N=178) | Pembrolizumab (N=69) | Sintilimab (N=39) | Tislelizumab (N=28) | P value |
| --- | --- | --- | --- | --- | --- | --- |
| R0 Resection (%) | R0 | 157 (88.2) | 65 (94.2) | 37 (94.9) | 26 (92.9) | 0.348 |
|  | R1 | 21 (11.8) | 4 (5.8) | 2 (5.1) | 2 (7.1) |  |
| PCR (%) | YES | 56 (31.5) | 17 (24.6) | 13 (33.3) | 4 (14.3) | 0.215 |
|  | NO | 122 (68.5) | 52 (75.4) | 26 (66.7) | 24 (85.7) |  |
| MPR (%) | YES | 97 (54.5) | 37 (53.6) | 21 (53.8) | 11 (39.3) | 0.514 |
|  | NO | 81 (45.5) | 32 (46.4) | 18 (46.2) | 17 (60.7) |  |
| TRG score (%) | TRG1 | 61 (34.3) | 22 (31.9) | 13 (33.3) | 5 (17.9) | 0.641 |
|  | TRG2 | 36 (20.2) | 15 (21.7) | 8 (20.5) | 6 (21.4) |  |
|  | TRG3 | 42 (23.6) | 11 (15.9) | 7 (17.9) | 10 (35.7) |  |
|  | TRG4 | 35 (19.7) | 18 (26.1) | 10 (25.6) | 5 (17.9) |  |
|  | TRG5 | 4 (2.2) | 3 (4.3) | 1 (2.6) | 2 (7.1) |  |
| Residual tumor pattern (%) | I | 47 (45.6) | 19 (45.2) | 8 (38.1) | 7 (35.0) | 0.595 |
|  | II | 10 (9.7) | 0 (0.0) | 1 (4.8) | 2 (10.0) |  |
|  | III | 9 (8.7) | 5 (11.9) | 4 (19.0) | 3 (15.0) |  |
|  | IV | 37 (35.9) | 18 (42.9) | 8 (38.1) | 8 (40.0) |  |
| PCR of LNM (%) | YES | 83 (55.3) | 29 (52.7) | 16 (55.2) | 9 (42.9) | 0.753 |
|  | NO | 67 (44.7) | 26 (47.3) | 13 (44.8) | 12 (57.1) |  |
| Total postoperative complications (%) | Yes | 71 (39.9) | 25 (36.2) | 10 (25.6) | 5 (17.9) | 0.072 |
| Major postoperative complications (%) | Yes | 17 (9.6) | 10 (14.5) | 2 (5.1) | 2 (7.1) | 0.408 |
| Clavien-Dindo grade (%) | I | 28 (39.4) | 9 (36.0) | 3 (30.0) | 1 (20.0) | 0.686 |
|  | II | 26 (36.6) | 6 (24.0) | 5 (50.0) | 2 (40.0) |  |
|  | III | 14 (19.7) | 7 (28.0) | 2 (20.0) | 2 (40.0) |  |
|  | IV | 3 (4.2) | 3 (12.0) | 0 (0.0) | 0 (0.0) |  |
| 30-d mortality (%) | Yes | 0 (0.0) | 0 (0.0) | 0 (0.0) | 0 (0.0) | -- |

PCR, Pathological complete response; MPR, Major pathological response; TRG, Tumor regression grade; LNM, Lymph node metastasis.
